# Dynamics of Soil Water Evaporation during Soil Drying: Laboratory Experiment and Numerical Analysis

**DOI:** 10.1155/2013/240280

**Published:** 2013-12-30

**Authors:** Jiangbo Han, Zhifang Zhou

**Affiliations:** School of Earth Sciences and Engineering, Hohai University, Nanjing 210098, China

## Abstract

Laboratory and numerical experiments were conducted to investigate the evolution of soil water evaporation during a continuous drying event. Simulated soil water contents and temperatures by the calibrated model well reproduced measured values at different depths. Results show that the evaporative drying process could be divided into three stages, beginning with a relatively high evaporation rate during stage 1, followed by a lower rate during transient stage and stage 2, and finally maintaining a very low and constant rate during stage 3. The condensation zone was located immediately below the evaporation zone in the profile. Both peaks of evaporation and condensation rate increased rapidly during stage 1 and transition stage, decreased during stage 2, and maintained constant during stage 3. The width of evaporation zone kept a continuous increase during stages 1 and 2 and maintained a nearly constant value of 0.68 cm during stage 3. When the evaporation zone totally moved into the subsurface, a dry surface layer (DSL) formed above the evaporation zone at the end of stage 2. The width of DSL also presented a continuous increase during stage 2 and kept a constant value of 0.71 cm during stage 3.

## 1. Introduction 

Evaporation from a porous medium plays a key role in various fields, including hydrological, agricultural, environmental, and engineering applications, such as water evaporation from soil surfaces and its application in hydrological modeling [1–4], salt accumulation in the near-surface layer [5, 6], food processing and preservation [7], production of ceramics and paper, eye and skin care, and a number of construction activities [8]. Soil evaporative drying is involved in coupled heat and mass transfer and depends on transport properties of liquid water, vapor and heat, atmospheric evaporative demand, and vapor and heat exchanges between the land surface and the atmosphere. A number of researches have been conducted to better understand drying behavior of soil or other porous media in past decades [6, 9–14].

Soil drying by evaporation has been often separated into three or two stages: the high rate (stage 1), the falling rate (stage 2), and/or the low and constant rate stages (stage 3) [15–19]. Since stages 2 and 3 evaporation both occur in the subsurface, the so-called stage 3 evaporation sometimes is incorporated into stage 2 evaporation. During stage 1, evaporation occurs at the soil surface and is limited by the atmospheric evaporative demand. With the progressive drying, when the surface moisture is depleted, the evaporation rate drops below the potential rate; then stage 2 or falling rate evaporation begins. During this stage, the location of evaporation shifts from surface to subsurface, resulting in formation of a dry surface layer (DSL). The subsurface evaporation is controlled by soil properties and mainly occurs in a narrow evaporation zone at the bottom boundary of the DSL [20]. The formation of DSL also has a significant impact on surface energy balance since the energy used for vaporization must be transported from the soil surface to the evaporation zone. The evaporation zone divides the soil into two parts, with only vapor flow occurring in the profile above the evaporation zone and liquid water flows mainly occurring in the profile below it [21, 22]. In the field condition, Novak [23] further recognized that the near-surface evaporation zone displayed a strong diurnal pattern superimposed on changes associated with the progressive drying of the soil. In addition, there are a few direct measurement approaches to describe the soil water evaporation. For instance, Heitman et al. [24, 25] developed a sensible heat balance approach to determinate in situ soil water evaporation dynamics by using heat-pulse probes. However, this approach could result in underestimation of total subsurface evaporation due to the existence of undetectable zone [26]. Deol et al. [12] further used the sensible heat balance approach to quantify the millimeter-scale subsurface evaporation profiles by using eleven-needle heat pulse probes in a soil column over a drying event. These measurement-based methods greatly improved our knowledge on the dynamics of evaporation zone. However, there were possibly some uncertainties in the accuracy of the shape and variation of evaporation zone, particularly for some cases whereas the width of evaporation zone was less than 1 mm [23, 26]. Furthermore, in addition to the evaporation zone, and DSL, the complete subsurface evaporation rate profile should include the condensation zone located “below” the evaporation zone, which was not often presented in recent numerical simulations or measurement approaches [12, 23, 26].

The objective of this study was to obtain complete dynamic information on soil water evaporation process during a continuous drying event, including the structures of evaporation zone, condensation zone and DSL in the soil profile and their evolutions with the increasing drying of the soil. To do this, an open soil column experiment was conducted under a radiation boundary to mimic the continuous drying of the soil. The numerical simulation then was carried out to evaluate the dynamics of soil water evaporation during the soil drying by using the coupled heat and water transfer model, which was first developed by Philip and de Vries [27], henceforth PDV, and then had been slightly modified in past decades [7–10]. The movement of liquid water and water vapor, driven by both pressure head and temperature gradients, and the movement of soil heat by conduction, convection of sensible heat by liquid water and water vapor flow, and transfer of latent heat by diffusion of water vapor were included in the model. The constant water table condition was also considered in the soil column experiment since, in field conditions, shallow groundwater conditions are often found in humid or semihumid climates or could be found in arid or semiarid environments where there were extensive irrigated areas.

## 2. Materials and Methods

### 2.1. Experimental Setup and Procedure

**Figure 1:**
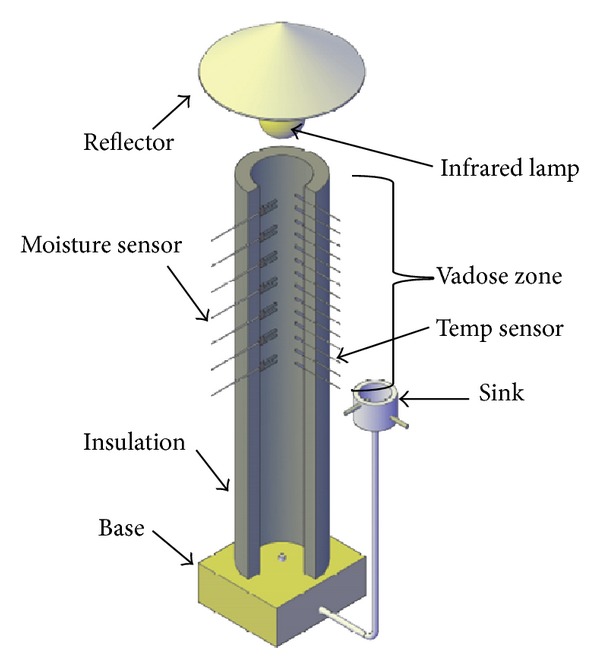
The schematic layout of the soil column apparatus as well as the layout of sensors within the column.

The vertical soil column (Figure 1), made from polymethyl methacrylate, had 150 cm in height, 0.5 cm in wall thickness, and an internal diameter of 20 cm, fastened with a 10 cm height wood material base at its bottom. The soil surface was located below the top of soil column with a distance of 5 cm. Water content and temperature distributions in the vadose zone were continuously monitored at 5 min intervals using dielectric soil moisture sensors (EC-5 Soil Moisture Sensor, Decagon Devices, Inc.) and temperature sensors (12 Bit Temperature Smart Sensor, Onset Computer Corp.), respectively. The air relative humidity above the column was obtained by using relative humidity sensor (12 Bit Temp/RH Smart Sensor, Onset Computer Corp.). Through the column walls, 14 temperature and 7 soil moisture sensors were installed radially at the center of soil column every 5 and 10 cm, respectively. The top temperature and moisture sensors were placed at the soil surface (barely covered with soil) and the depth of 5 cm below the soil surface, respectively. There were other temperature sensors used to monitor temperatures in the saturated zone and air which were not shown in Figure 1. All sensors were connected to the data logger system (HOBO Weather Station-H21-001, Onset Computer Corp.) for automatic data recording.

The physical parameters of the sand sample used in this experiment were summarized in Table 1. The sand was poured into the column in 2 cm increments with an effort to achieve a uniform soil density. In this procedure, maximum densities were achieved by the repeated and thorough tapping of the column wall. Smits et al. [2] reported that this procedure could provide greater densities than the use of a vibratory device which could damage the network of sensitive sensors. In order to minimize the radial heat loss, the rubber and plastic insulation materials were used to reduce ambient temperature interference and to produce approximately one-dimensional temperature distributions in soils.

The bottom of the soil column was connected to a sink to maintain a constant water table at 65 cm below the soil surface. The water table of the sink came from the continuous supply of a peristaltic pump (not shown). The desired water table was obtained by adjusting the height of water sink. In this procedure, the water table was initially established near the top soil surface of the column (fully saturated), then adjusting the height of external sink to the depth of 65 cm below the soil surface to produce a naturally drained vadose zone profile. Before the beginning of the experiment, the top soil surface was covered with a plastic sheet to reduce evaporation. The infrared lamp was hanged above the soil surface with a distance of 15 cm. When the experiment started, the plastic sheet was removed, and then the infrared lamp and the peristaltic pump were turned on. Temperature and water content data were then recorded by using the data acquisition system for 36 hours. The air temperature in laboratory was maintained at 8 ± 2 (°C) and assumed no effect on the heat transfer process in the soil column.

### 2.2. Models

#### 2.2.1. Liquid Water and Water Vapor Flows

Based on the PDV model [27], the flux densities of liquid water *q*_*L*_ (m s^−1^) and water vapor *q*_*v*_ (expressed as an equivalent water flux density m s^−1^) are given by, respectively,

(1) $$\begin{array}{c} q_{L}=q_{TL}+q_{hL}=-D_{TL}\frac{\partial T}{\partial z}-K\left(\frac{\partial h}{\partial z}+1\right), \end{array}$$

(2) $$\begin{array}{c} q_{v}=q_{Tv}+q_{hv}=-D_{Tv}\frac{\partial T}{\partial z}-D_{hv}\frac{\partial h}{\partial z}, \end{array}$$

where *q*_*TL*_ and *q*_*hL*_ are the thermal and isothermal liquid water fluxes (m s^−1^), respectively; *q*_*Tv*_ and *q*_*hv*_ are the thermal and isothermal water vapor fluxes (m s^−1^), respectively; *T* is the temperature (°C); *h* is the pressure head (m); *z* is the spatial coordinate positive upward (m); *D*_*TL*_ (m^2^ K^−1^ s^−1^) and *K* (m s^−1^) are the thermal and isothermal hydraulic conductivities for liquid phase fluxes, respectively; and *D*_*Tv*_ (m^2^ K^−1^ s^−1^) and *D*_*hv*_ (m s^−1^) are the thermal and isothermal vapor hydraulic conductivities, respectively. Based on the mass conservation, a general partial differential equation for describing the transient water flow under variably saturated, nonisothermal conditions can be expressed as

(3) ∂θ∂t=−∂qw∂z=−∂qL∂z−∂qv∂z=∂∂z[DTL∂T∂z+K∂h∂z+DTv∂T∂z+Dhv∂h∂z+K],

where *θ* is the total volumetric water content (m^3^ m^−3^), *q*_*w*_ (equal to the sum of liquid water and vapor densities) is the total water flux (m s^−1^), and *t* is time (s). The total volumetric water content is defined as

(4) $$\begin{array}{c} \theta =\theta_{L}+\theta_{v}, \end{array}$$

where *θ*_*L*_ is liquid volumetric water content (m^3^ m^−3^), *θ*_*v*_ is water vapor content (expressed as an equivalent water content, *ρ*_*v*_ *θ*_air_/*ρ*_*L*_, m^3^ m^−3^), *θ*_air_ is the volumetric air content (m^3^ m^−3^), and *ρ*_*L*_  ( = 1000 − 7.3 × 10^−3^(*T*−4)^2^ + 3.79 × 10^−5^(*T*−4)^3^) is the density of liquid water (kg m^−3^) at *T* (°C).

The mass continuum equation (3) can be divided into two equations that account for liquid water and water vapor contents in the soil, respectively:

(5) $$\begin{array}{c} \frac{\partial \theta_{L}}{\partial t}=-\frac{\partial q_{L}}{\partial z}-e, \end{array}$$

(6) $$\begin{array}{c} \frac{\partial \theta_{v}}{\partial t}=-\frac{\partial q_{v}}{\partial z}+e, \end{array}$$

where *e* is the depth-dependent evaporation or condensation rate (s^−1^) and *E*_*i*_  ( = *edz*) is the subsurface evaporation rate (m s^−1^) at each numerical node in the soil column (positive for evaporation and negative for condensation) [26, 28].

#### 2.2.2. Soil Hydraulic Properties

The accurate descriptions of soil hydraulic properties are essential to predict the behavior of water flow in the unsaturated zone, especially when the soil was drying with low water contents, the film flow becomes the dominant mechanism in the liquid flow, which is distinct with the capillary flow at a high water content. Fayer and Simmons [29] proposed an unsaturated hydraulic property model to better represent the soil water retention curve and unsaturated hydraulic conductivity at low water contents, which has been well applied in some previous researches [26, 28]:

(7) $$\begin{array}{c} \theta_{L}=\chi \theta_{a}+\left(\theta_{s}-\chi \theta_{a}\right){\left[1+{\left(-\alpha h\right)}^{n}\right]}^{-(1-1/n)}, \end{array}$$

(8) $$\begin{array}{c} K=K_{s}S_{e}^{l}{\left[\frac{\int_{0}^{S_{e}}dS_{e}/\left|h\right|}{\int_{0}^{1}dS_{e}/\left|h\right|}\right]}^{2}, \end{array}$$

where *θ*_*s*_ is saturated water content (m^3 ^m^−3^); *α* (m^−1^), *n* (dimensionless), and *θ*_*a*_ (m^3 ^m^−3^) are empirical shape parameters; *χ* is described as 1 − ln⁡(|*h*|)/ln⁡(|*h*_*m*_|), where *h*_*m*_ is the pressure head at the water content equal to 0 and is generally taken to be −10^7^ cm [29]; *K*_*s*_ is the saturated hydraulic conductivity (m s^−1^); *S*_*e*_  ( = *θ*_*L*_/*θ*_*s*_) is the effective liquid saturation (dimensionless); and the pore connectivity coefficient, *l*, was given a value of 0.5 as suggested by Mualem [30]. Soil water retention curves measured in the laboratory were fitted to (7) and the resulting parameters could be seen in Table 1.

The thermal hydraulic conductivity for the liquid flux, *D*_*TL*_, is defined as [31]

(9) $$\begin{array}{c} D_{TL}=K\left(hG_{wT}\frac{1}{\gamma_{0}}\frac{\partial \gamma }{\partial T}\right), \end{array}$$

where *G*_*wT*_( = 7) is the gain factor (dimensionless), which corrects the temperature dependence of the surface tension [32], and *γ*_0_ ( = 71.89 g s^−2^) and *γ*  ( = 75.6 − 0.1425*T* − 2.38 × 10^−4^ *T* ^2^ (g s^−2^)) are the surface tension of soil water at 25 (°C) and *T* (°C), respectively. The thermal (*D*_*Tv*_) and isothermal (*D*_*hv*_) vapor hydraulic conductivities are described by Heitman et al. [33], respectively:

(10) $$\begin{array}{c} D_{Tv}=\frac{D}{\rho_{L}}\eta \left[H_{r}\frac{d\rho_{sv}}{dT}+\rho_{sv}\frac{dH_{r}}{dT}\right], \end{array}$$

(11) $$\begin{array}{c} D_{hv}=\frac{D}{\rho_{L}}H_{r}\frac{Mg}{RT}\rho_{sv}, \end{array}$$

where *D* is the vapor diffusivity in soil (m^2^ s^−1^), *η* is the enhancement factor (dimensionless), *H*_*r*_ is the relative humidity (dimensionless), *ρ*_*sv*_  ( = 10^−3^ × exp⁡(19.84 − 4975.9/(*T* + 273.15))) is the saturated vapor density (kg m^−3^) at *T* (°C), *M* ( = 0.01805 kg mol^−1^) is the molecular weight of water, *g* ( = 9.81 m s^−2^) is the gravitational acceleration, and *R* ( = 8.314 J mol^−1^ K^−1^) is the universal gas content. The diffusivity of water vapor *D* in soil is described as

(12) $$\begin{array}{c} D=D_{a}\Omega \theta_{\text{air}}, \end{array}$$

where *D*_*a*_( = 2.12 × 10^−5^(*T*_abs_/273.15)) is the diffusivity of water vapor in air (m^2^ s^−1^), *T*_abs_ is absolute temperature (K), and *Ω*  ( = *θ*_air_^2/3^) is the tortuosity factor (dimensionless) described as a function of air content [34]. The relative humidity, *H*_*r*_, (dimensionless) can be described by using a thermodynamic relationship between liquid water and water vapor in soil pores [27]:

(13) $$\begin{array}{c} H_{r}=\exp\left(\frac{hMg}{RT}\right) \end{array}$$

and the enhancement factor *η* (dimensionless) described by Cass et al. [35]:

(14) $$\begin{array}{c} \eta =a+3\frac{\theta_{L}}{\theta_{s}}-\left(a-1\right)\exp\left\{-{\left[\left(1+\frac{2.6}{\sqrt{f_{c}}}\right)\frac{\theta_{L}}{\theta_{s}}\right]}^{4}\right\}, \end{array}$$

where *a* is an empirical constant to be fitted in this study and *f*_*c*_ was the mass fraction of clay in the soil.

#### 2.2.3. Heat Transport and Soil Thermal Properties

The soil heat flux density *q*_*h*_ (J m^−2^ s^−1^), accounting for the sensible heat of the conduction, sensible heat by the convection of liquid water and water vapor, and latent heat by vapor flow, can be described as

(15) $$\begin{array}{c} q_{h}=-\lambda \frac{\partial T}{\partial z}+C_{v}\left(T-T_{r}\right)q_{v}+C_{w}\left(T-T_{r}\right)q_{L}+L_{0}q_{v}, \end{array}$$

where *λ* is the apparent soil thermal conductivity (W m^−1 ^K^−1^) described by Chung and Horton [36]:

(16) $$\begin{array}{c} \lambda =b_{1}+b_{2}\theta_{L}+b_{3}{\left(\theta_{L}\right)}^{0.5}, \end{array}$$

where *b*_1_, *b*_2_, and *b*_3_ are empirical parameters (W m^−1 ^K^−1^) (for sand: *b*_1_ = 0.228, *b*_2_ = −2.046, *b*_3_ = 4.909); *C*_*w*_ ( = 4.18 MJ m^−3^ K^−1^) and *C*_*v*_ ( = 1.87 MJ m^−3^ K^−1^) are the volumetric heat capacities of liquid water and water vapor, respectively; *L*_0_  ( = *ρ*_*L*_∗(2.501 × 10^6^ − 2369.2*T*)) is the volumetric latent heat of vaporization of water (J m^−3^); and *T*_*r*_ is the arbitrary reference temperature (°C). The storage of heat *S*_*h*_ (J m^−3^) in the soil is

(17) $$\begin{array}{c} S_{h}=C_{s}\left(T-T_{r}\right)\theta_{n}+C_{v}\left(T-T_{r}\right)\theta_{v}+C_{w}\left(T-T_{r}\right)\theta_{L}+L_{0}\theta_{v}, \end{array}$$

where *C*_*s*_ ( = 1.92 MJ m^−3^ K^−1^) is the volumetric heat capacities of dry soil particles and *θ*_*n*_ is the volumetric fraction of solid phase (m^3^ m^−3^). The continuity equation for the conservation of energy in a variably saturated rigid porous medium is

(18) $$\begin{array}{c} \frac{\partial S_{h}}{\partial t}=-\frac{\partial q_{h}}{\partial z}-Q, \end{array}$$

where *Q* is the energy sources or sinks (J m^−3^ K^−1^). Combining the continuity equation with (15) and (16) produces the governing equation for the movement of energy in soil [37]:

(19) Cp∂T∂t+L0∂θv∂t+Cv(T−Tr)∂θv∂t+Cw(T−Tr)∂θL∂t  =∂∂z(λ∂T∂z)−Cv∂Tqv∂z−Cw∂TqL∂z−L0∂qv∂z   −∂∂z(hT∗(T0−T)),

where *C*_*p*_  ( = *C*_*s*_ *θ*_*n*_ + *C*_*v*_ *θ*_*v*_ + *C*_*w*_ *θ*_*L*_) represents the volumetric heat capacity of the moist soil (J m^−3^ K), the contribution of air to *C*_*p*_ is considered negligible, the last term on the right of (19) represents the lateral heat loss term which accounts for the energy flux through column walls, *h*_*T*_ ( = 12 J s^−1^ m^−2^ K^−1^) is the total energy transfer coefficient determined by the other experiment using the heat transfer method [38], and *T*_0_ is the initial temperature value.

### 2.3. Initialization and Boundary Conditions

The soil domain of interest covered the entire vadose zone from the soil surface to the water table at 65 cm below the soil surface. Initial soil temperatures and water contents in the soil column were collected by using sensors, both of which were fitted as polynomial functions of depth for the use in the simulation. A time function boundary condition for temperature was assigned at the top boundary using measured temperature values, and temperature was fixed at 8 (°C) at water table. The temperature of water from the sink was assumed to be equal to the air temperature during the simulation period. The surface boundary condition for water was expressed as

(20) $$\begin{array}{c} q_{L}\left(0,t\right)+q_{v}\left(0,t\right)=E_{s}, \end{array}$$

where *E* is surface evaporation rate (m s^−1^), calculated from the difference between the water vapor densities of the air, *ρ*_*va*_ (kg m^−3^), and the soil surface, *ρ*_*vs*_ (kg m^−3^):

(21) $$\begin{array}{c} E=\frac{\rho_{vs}-\rho_{va}}{r_{s}*\rho_{L}}=\frac{H_{rs}*\rho_{sv}-H_{r\text{air}}*\rho_{sv}}{r_{s}*\rho_{L}}, \end{array}$$

where *r*_*s*_ is the soil surface resistance to water vapor flow (s m^−1^), *H*_*rs*_ is relative humidity at the soil surface, calculated by using (13), and *H*_*r*air_ is the relative humidity above the column, measured by using the relative humidity sensor. Since wind resistance and surface roughness were considered negligible in the laboratory, aerodynamic resistance to water vapor transfer was ignored in this study. Bittelli et al. [3] suggested that the van de Griend and Owe [39] model could provide the best estimates of evaporation. And this model can be expressed as

(22) $$\begin{array}{c} r_{s}=10*\exp\left(35.63\left(\theta_{rw}-\theta_{\text{top}}\right)\right), \end{array}$$

where *θ*_*rw*_ is an empirical parameter (m^3^ m^−3^) and *θ*_top_ is the water content (m^3^ m^−3^) at the near-surface zone. At the bottom boundary of the vadose zone, the water content is fixed as saturated water content *θ*_*s*_.

### 2.4. Numerical Simulation

The soil water and energy governing equations subject to the boundary and initial conditions were solved using a commercial implementation (COMSOL Multiphysics, Version 4.2a) of the finite element method. Because mesh densities possibly affected the simulation results [23, 26], the mesh was refined many times to achieve a maximum density in order to minimize numerical oscillations and differences in results as compared with different mesh-sized simulations. The 65 cm long soil profile was divided into 1728 elements with a thickness of 0.03762 cm and this fine element thickness ensured the adequate numerical resolution to obtain the solution mathematically. Soil temperatures and water contents for each discretized node were output in 100-second intervals.

Each parameter required for the numerical simulation was either derived from the previously mentioned expressions or obtained through measurements. Because the enhancement factor was a parameter of all-inclusive mechanics to explain the disagreement between observed and calculated data in the PDV model, the parameter *a* in the enhancement factor expression (14) was chosen as the only parameter to calibrate the model. The calibration process was to set an objective function of the observed and calculated water contents and to minimize it to obtain the optimum value, which was similar to the process used by Sakai et al. [26, 28]. A resulting value of *a* was 2.3, which was used for the implementation in the numerically coupled equations governing liquid water, water vapor, and heat transport.

## 3. Results and Discussion

### 3.1. Observed and Simulated Soil Temperatures and Water Contents

**Figure 2:**
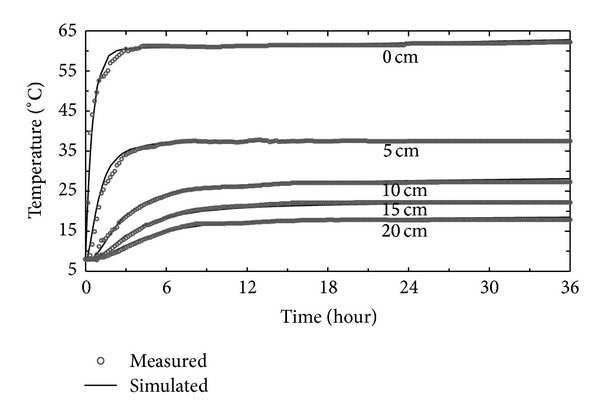
Temporal changes in simulated and measured soil temperatures at different depths.

Figure 2 shows temporal variations in observed and calculated soil temperatures at selected depths of 0, 5, 10, 15, and 20 cm during a 36 h period. It was clear that simulated soil temperatures at all depths compared well with measured values during the entire simulation period. The soil surface (0 cm) temperature increased rapidly within about 3 h, thereafter increasing slowly and gradually approaching a constant value, indicating that a heat balance between the soil system and external environment formed after 3 hours. Soil temperatures at other depths also present temporal trends similar to temperature at the soil surface. Because of attenuation of the heat energy transported from the surface, the temperature typically decreased with depth.

**Figure 3:**
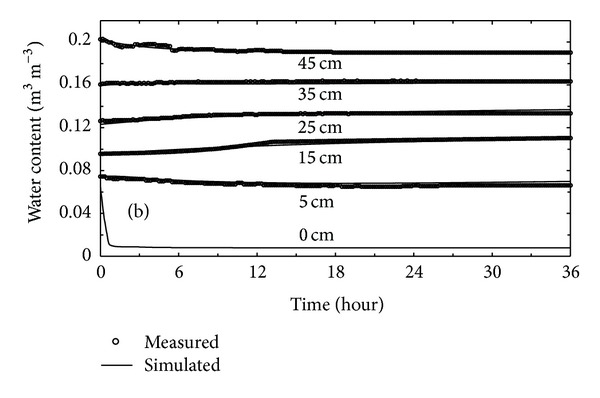
Temporal changes in simulated and measured soil water contents at different depths.

The temporal variations in the volumetric soil water content at selected depths during the experiment period are shown in Figure 3. Simulated soil water contents follow well with measured values at all depths, indicating that the calibrated model can also catch temporal variations of the soil water content. Because water content obtained by moisture sensor just represented a specified volumetric range of soil water content, soil moisture sensor was not suitable to be placed very close to the soil surface. As shown in Figure 3, the simulated water content at soil surface decreased rapidly during 0.6 h and then kept a nearly constant value. Observed and calculated water contents at depths of 5 and 45 cm, at the upper and lower parts of profile, respectively, also decreased but more gradually over time. However, both simulated and measured water contents at the depths of 15 and 25 cm exhibited small increase during the simulation period, which resulted from the condensation of water vapor. And the water content at the 35 cm depth remained nearly constant during the simulation period.

### 3.2. Surface Liquid Water and Vapor Fluxes

**Figure 4:**
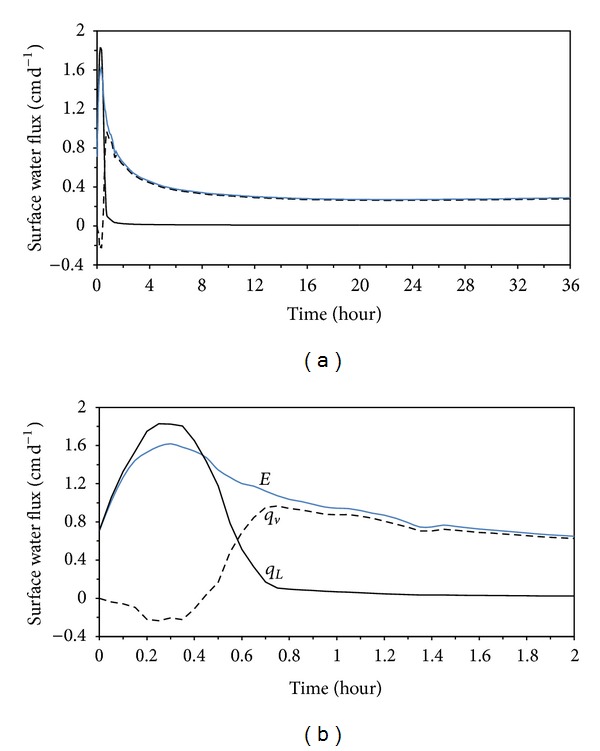
Calculated surface liquid-fluxes *q*_*L*0_ (solid line), vapor-fluxes *q*_*v*0_, (dashed line), and surface evaporation rate *E* (blue solid line) during whole simulation period (a). (b) is plotted during 2 hours for the emphasis of the early periods of soil drying.

The calculated temporal evolution in surface liquid-water *q*_*L*0_  ( = *q*_*L*_(0, *t*)) and water-vapor fluxes *q*_*v*0_ ( = *q*_*v*_(0, *t*)) and surface evaporation rate *E* (equal to the sum of *q*_*L*0_ and *q*_*v*0_) as described in (20) are illustrated in Figure 4. *q*_*L*0_ represents the process of surface evaporation in which liquid water moved from the deeper profile and then vaporized at the soil surface, while *q*_*v*0_ indicates the process of subsurface evaporation in which liquid water vaporized below the soil surface and the produced water vapor moved toward the soil surface. As shown in Figure 4(b), stage 1 evaporation maintained about 0.6 h, and characterized a relatively large surface evaporation rate *E*. The surface evaporation rate *E* was dominated by *q*_*L*0_ during this period, indicating that evaporation was occurring at the soil surface. A short period from about 0.6 to 1.2 h could be viewed as the transition of evaporation from stage 1 to stage 2, in which *E*_*s*_ was contributed by both *q*_*L*0_ and *q*_*v*0_. That is, both surface and subsurface evaporation occurred simultaneously during this transient stage. Stage 2 evaporation, also referred to as the falling-rate or soil-limited evaporation stage, began at about 1.2 h during which the evaporation rate dropped below the potential rate and had a continuous decrease with the increasing drying of the soil. During this stage the value of *q*_*L*0_ was close to 0 and *q*_*v*0_ became the only source of *E*_*s*_. Stage 3 evaporation, which was characteristic of a very low and constant evaporation rate, could be observed after about 16 h in Figure 4. Note that, before about 0.5 h, *q*_*v*0_ in Figure 4 presents the negative values, indicating that some water vapor generated at the soil surface would diffuse into the subsurface from the soil surface. This process resulted from the effect of the downward temperature gradient near the soil surface.

### 3.3. Dynamics of Phase Change Zone

**Figure 5:**
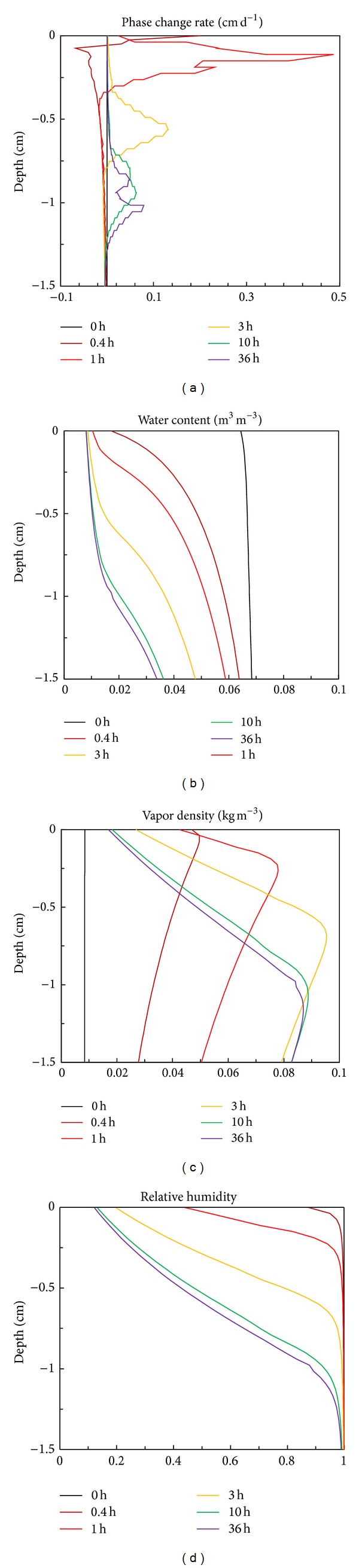
(a) Subsurface evaporation rate, (b) water content, (c) vapor density, and (d) relative humidity profiles at selected times above 1.5 cm depth.

Figure 5 presents the simulated subsurface phase change rate *E*_*s*_, water content, vapor density, and relative humidity in the 1.5 cm profile at different times. The subsurface evaporation or condensation rate, *E*_*s*_, represents the phase change rate between liquid water and water vapor at each calculation node within the soil [20, 26]. According to (6), *E*_*s*_ in the simulation can be calculated from

(23) $$\begin{array}{c} E_{i}^{j}=\left(\theta_{vi}^{j}-\theta_{vi}^{j-1}\right)\frac{dz}{dt}+\left(q_{vi+1/2}^{j-1/2}-q_{vi-1/2}^{j-1/2}\right), \end{array}$$

where the subscripts *i* and *j* represent the indexes of depth and time steps, respectively. *E*_*s*_ was depth dependent and different from the surface evaporation rate *E*, and the latter represented the summation of subsurface evaporation. The evaporation zone was the region where *E*_*s*_ was greater than 0 in Figure 5(a), while the region where *E*_*s*_ was less than 0 was referred to as the condensation zone which was always located immediately below the evaporation zone. The boundary between the evaporation and condensation zones generally corresponded with the peak of vapor density (Figure 5(c)) and the inflection of relative humidity (Figure 5(d)). According to Fick's vapor diffusion law, water vapor will diverge from the peak of vapor density. Thus, a part of water vapor driven by the pressure head gradient would move upward from the peak to the soil surface. And another part of water vapor moved downward by the temperature gradient, and when it came across the cooler soil than the above profile, the water vapor was converted to liquid water within the condensation zone where the relative humidity was close to the value of 1 (Figure 5(d)).

The subsurface evaporation zone was a relatively narrow soil profile showing a normally distributed rate around the peak value, as shown in Figure 5(a). During stage 1, for example, at 0.4 h, evaporation occurred at the soil surface and the peak of subsurface evaporation rate was located at the surface. And at 1 h during the transition stage, except for a little evaporation still occurring at the soil surface, most of evaporation as well as the peak took place within the subsurface, indicating that a shift from surface to subsurface evaporation was occurring. With further drying of the soil, the evaporation zone totally moved into the subsurface at 3 h in Figure 5(a) and became deeper and wider from 3 to 36 h. There was a sharp decrease in the water content within the evaporation zone where liquid water was changed to vapor (Figure 5(b)). Above the evaporation zone, the water content reached its critical values and the near-surface profile was approximately air-dry. The condensation rate was relatively small and its peak decreased with time (absolute value). However, the actual width of condensation zone could extend from the bottom boundary of evaporation zone to the deeper profile, which was contributed to some water storage at the middle part of vadose zone. This could be found in Figure 2 which showed that the water content at depths of 15 and 25 cm had a small increase with time.

**Figure 6:**
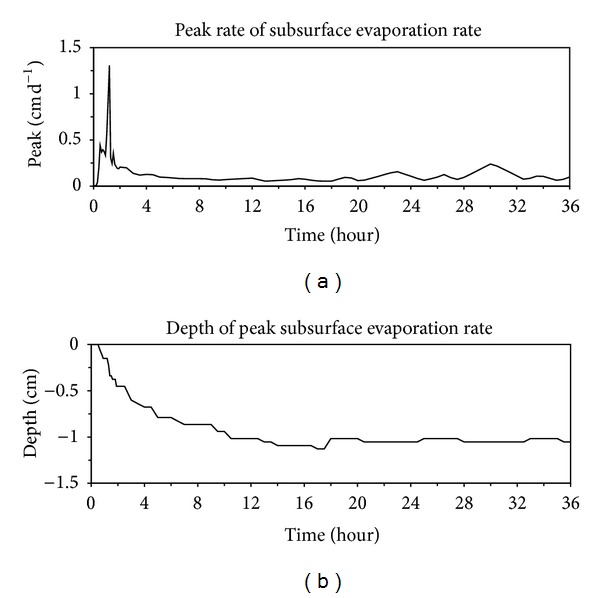
Temporal changes in peaks of the subsurface evaporation rate (a) and its locations (b) in the subsurface evaporation rate profile.

**Figure 7:**
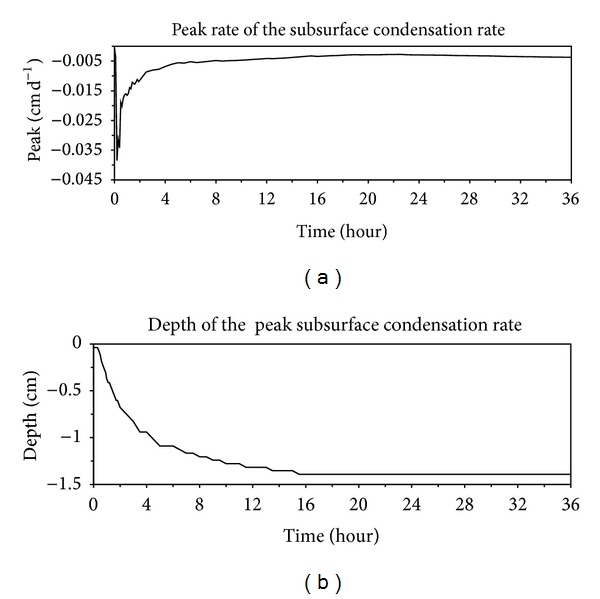
Temporal changes in peaks of subsurface condensation rate (a) and its locations (b) in the subsurface evaporation rate profile.

Calculated temporal changes in peaks of the subsurface evaporation rate are shown in Figure 6(a). There was an increasing trend in the peak from 0 to 0.6 h when it was located at the soil surface during stage 1 (e.g., 0.4 h in Figure 5(a)), followed by a sharp increase from 0.6 to 1.2 h during the transition stage when peak occurred in the subsurface (e.g., 1 h in Figure 5(a)). After the evaporation zone totally moved into the soil, the peak value decreases rapidly during the early period of stage 2 evaporation and then kept nearly constant during stage 3. The peak of the condensation rate presents the same trend as that of subsurface evaporation rate, but with negative and smaller values (Figure 7(a)). The locations of peaks of evaporation rate and condensation rate in the soil profile (Figures 6(b) and 7(b)) deepened with time until about 16 h when stage 3 evaporation began. During stage 3, depths of the two peaks remained nearly constant with time, with the peak of condensation rate occurring deeper in the profile.

**Figure 8:**
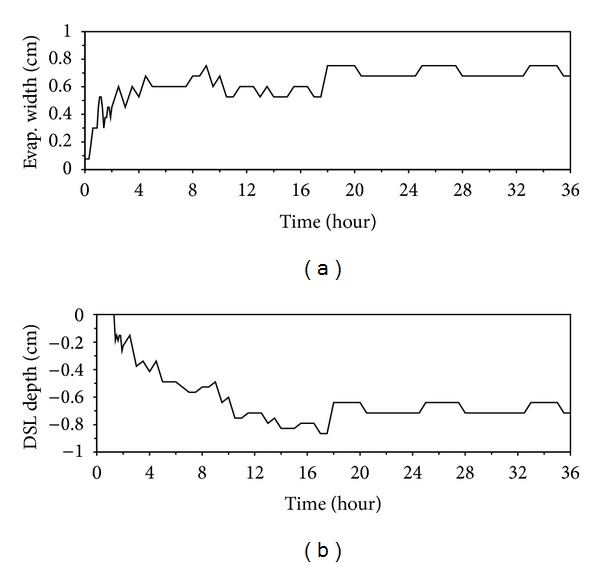
Temporal changes in the width of (a) the evaporation zone and (b) the depth of DSL in the profile.

The widths of evaporation zone and DSL could be determined from the subsurface evaporation rate profiles. Note that the bottom boundary of DSL was located above the evaporation zone [20]. Therefore, we used the location of the bottom boundary of DSL in the profile to represent its width.  Figure 8 presents the estimated temporal changes in the widths of evaporation zone and DSL. The noisy part in these curves in Figure 8 was due to numerical oscillation which typically occurred in the numerical simulation by using the finite element or finite difference method. The width of evaporation zone had a rapid increase during stage 2 and approached a relatively constant width of 0.68 cm during stage 3. During stage 1 and transition stage evaporation (from 0 to 1.2 h), the top boundary of the evaporation zone was at the soil surface and hence the width of DSL was 0, for example, at 0.4 h in Figure 5(a). When the top boundary of evaporation zone moved into the soil, the DSL formed above the evaporation zone. The thickness of DSL also increased rapidly during stage 2, and finally remained constant during stage 3 evaporation with a width of about 0.71 cm, suggesting that the downward development of DSL was restricted by the constant water supply from the water table at 65 cm in the present study.

## 4. Conclusions

To obtain a better understanding of dynamics of soil water evaporation during the soil drying, the soil column experiment was conducted in laboratory and then the numerical analysis on the evaporation process was carried out. The coupled heat and water transfer model based on the PDV theory was well calibrated by the observed water content and temperature data. Results show that soil drying by evaporation could be divided into three stages, beginning with a relative high evaporation rate during stage 1 evaporation, followed by a lower rate during transient and stage 2 and finally remaining a very low and constant rate during stage 3.

Stage 1 evaporation was very short, during which the peak of evaporation rate occurred at the soil surface. During the transition period from stage 1 to 2, this peak moved into the subsurface, but with the top boundary of the evaporation zone still at the soil surface. At the beginning of stage 2, when the top boundary also moved into the subsurface, the DSL began to form above the evaporation zone, with its width increasing during stage 2 and finally reaching a nearly constant value of 0.71 cm during stage 3. The constant width of DSL during stage 3 indicated that the development of DSL was finally restricted by the constant water supply from the shallow water table. The peaks of subsurface evaporation and condensation rates in the profile presented continuously increase during stage 1 and transition stage and then decreased during stage 2 and finally remained constant during stage 3, with the smaller values and deeper locations for peaks of the condensation rate. The width of evaporation zone kept a continuous increase during stage 1 and stage 2 and remained a nearly constant value of 0.68 cm during stage 3.

Although the magnitude of condensation zone was much smaller than that for the evaporation zone, its width was apparently wider than that of evaporation zone. This condensation process resulted in the water content increase in some depths in the profile (e.g., at depths of 15 and 25 cm). Therefore, this condensation zone was as important as the evaporation zone in the contribution to soil water dynamics and should be considered in future studies on soil drying processes.

## Figures and Tables

**Table 1:** Selected properties of sand sample.

Soil sample	Particle size range (mm)	Average particle size (mm)	Dry bulk density (g cm^−3^)	Porosity	Saturated hydraulic conductivity *K*_*s*_ (m s^−1^)	Fayer and Simmons model parameters		
						*θ*_*a*_	*α* (cm^−1^)	*n*
Sand	0.05–1	0.35	1.62	0.335	2.5 × 10^−5^	0.098	0.165	1.49
